# Introductory Address Delivered at the Opening of the Session, 1857-58

**Published:** 1858-01

**Authors:** B. G. Babington

**Affiliations:** President of the Society.


### TRANSACTIONS

OF THE

### EPIDEMIOLOGICAL SOCIETY OF LONDON.

### Papers and Communications.

### INTRODUCTORY ADDRESS, DELIVERED AT THE OPENING OF THE SESSION 1857-8.

By B. G. BABINGTON, M.D., F.R.S., President of the Society.

In the course of the brief address which I had the honour to deliver from this chair, at the opening meeting of last session, I took occasion to remark that, as peace had happily been restored to Europe, we might reasonably expect that the medical officers of the military services, the Sanitary Commissioners, and the medical men from the ranks of civil life who had been employed in the East, would at no distant time present us with the fruits of their experience in the ample field for observation which the recent campaign had afforded them.

That expectation has already to some extent been realised. The Sanitary Commissioners have published a lengthened and valuable report on the sanitary condition of our army in the Crimea, which has called forth a reply from Sir John Hall, the late head of the medical department of the army in the East. The results of the observation of several other civil as well as military medical officers, on the same field, have likewise appeared; and, at the present moment, at least two important works from the same source are announced as being about to issue from the press. We have, indeed, among the presentations to the library to night, an interesting monogram on "Cholera at Varna," etc., by Dr. Mackay, late surgeon of H.M. ship *Agamemnon* in the Black Sea.

I may also state, that the excellent paper by Dr. Smart, on the "Climate of the Crimea, and its Effects upon Health, as observed during the first year of the occupation by the Allied Forces;" as well as two communications, "On the Cholera Epidemy at Fogo in the Cape de Verds," by Josè Fernandez da Silva Leaō; and "On the History of Gaol Fever in England," by Dr. Webb, have been published among the *Transactions* of the Society in Dr. Richardson's Sanitary Review.

From the medical staff of our allies, also several important works have emanated, bearing upon the diseases, etc., of the French army during the eastern campaign. I may here allude to "A Report on the Sanitary State of the French Army," by M. Michel Lévy; and "Recherches sur les Maladies de l'Armée d'Orient pendant l'Hiver de 1854 à 1855," par M. le Docteur Tholozan.

It is but too painfully known to us that, since the close of the Russian war, and, indeed, only within the last few months, the mutiny of the greater portion of our native troops in India has again drawn heavily upon the military resources of this country. It is not my business here to enter into any detail of the unparalleled atrocities by which this rebellion has acquired, and will ever retain, so hideous a notoriety. I will only observe that our European troops, whether during the long and fatiguing marches to relieve our countrymen and countrywomen in beleaguered forts, or in the no less harassing duties in the Camp before Delhi (now happily in British possession), have had to encounter a far more formidable, and, if possible, a more treacherous foe than the sepoy. Havelock has won ten battles consecutively in the course of a few months—cholera is the only enemy before which he has ever retreated.

Cholera has also for some time been prevalent at Hamburgh, and in several of the Baltic ports; and within the last three weeks some undoubted cases of the same disorder have appeared at Stratford, in Essex; and one, which proved rapidly fatal, occurred about a fortnight back at Poplar, in the eastern district of London.

I am not aware that the disease has at all spread in these districts, or that it has broken out elsewhere in this country; but the proximity of so terrible a scourge is suggestive of the necessity of watching its movements, and taking measures to meet its appearance in an epidemic form.

The attention of our Cholera Committee will, I have no doubt, be forthwith directed to this matter, aware as they are of the great importance, when an outbreak occurs, of ascertaining the origin and history of first cases.

Those of you who have regularly attended the general meetings of the society, are aware that, during the past session, some very important papers have been read; and, according to custom, I now proceed to bring them again under your notice, by stating the titles of these communications.

On Monday, November 3rd, 1856, after a brief introductory address by the president, Dr. Milroy read a paper "On the Mode of Investigating Epidemic Diseases."

On Monday, December 1st, 1856, a paper "On the Climate of the Crimea, and its effects on Health, as observed during the first year of the occupation by the Allies," by William E. Smart, M.D., was read by Dr. McWilliam.

On Monday, March 2nd, 1857, Professor Murphy read the concluding portion of a paper "On Puerperal Fever."

On Monday, April 6th, 1857, a paper "On an Outbreak of Fever on board the Turkish Admiral's Ship at Sinope, and on Scurvy on board a Turkish Brig of War," by J. N. Radcliffe, Esq., late of the Ottoman Contingent, was read by Dr. McWilliam.

On Monday, May 4th, 1857, a paper "On the Cholera Epidemy at Fogo, in the Cape de Verds, in 1855," by Josè Fernandes da Silva Leaõ, was read by Dr. McWilliam; also, "Extract from the Report of Dr. Agostinho Josè Ramos de Carvalho, chief of the Medical Staff of the Cape de Verd Islands; "Observations on Cholera, as it appeared at Rio de Janeiro in 1855-6," by Dr. G. Lee, of that city; and a paper "On Cholera at Para, in the Brazils, in 1855-6," by Samuel Vines, Esq., H. B. M. Consul at Para.

On Monday, July 6th, 1857, a paper "On the History of Gaol Fever in England," by Dr. Webb, was read by the Author.

On Monday, August 3rd, 1857, a paper "On the Local Causes influencing the type and diffusion of Epidemics," by W. I. Cox, Esq., was read by Dr. McWilliam.

The painful duty devolves on me on this occasion to announce to you that Mr. Tucker, the founder of our society and one of our honorary secretaries, has been removed from among us by a mental disease of the most distressing kind. I sincerely believe that his illness was at least much aggravated, if not altogether caused, by his unremitting exertions in our behalf. For this object he, indeed, postponed attention to his own business, and the consequence was, that when overtaken by the disease with which he is still afflicted, his affairs were in no flourishing condition. You will, I am sure, be gratified to learn that a very considerable amount of subscription has been raised for him among his friends, as well of this society as of the profession generally, and we are not without hope of being able thus to make a permanent, though it may not be an ample provision, for his future support.

Before sitting down, I am desirous to call the attention of the society to the "National Association for the Promotion of Social Science," which, as you are aware, was last month instituted at Birmingham, under the presidency of the veteran Lord Brougham.

The plan by which the association aims at the attainment of its object, is "to bring together, once in every year, the various societies and individuals who are interested in the subject, for the mutual interchange of reliable information, and the discussion of the great social problems of the day."

Public Health formed one of the five departments into which the subjects for consideration at the Birmingham meeting were divided; and a deputation from this Society, consisting of two members of the Council, attended the sittings of that Department, which was presided over by Lord Stanley, a nobleman who has already earned an honourable name in connection with the advancement of human welfare.

These gentlemen, in conformity with a resolution of our Council, were prepared with a report of the objects and proceedings of our Society, to be read before a meeting of the Public Health Department. No pains were spared, on the part of those who undertook to draw it up, to render this report effective; for it was thought right to inform the Association how actively we had for years been engaged in the endeavour to promote the cause of sanitary science and of the public health. So abundant, however, were the materials brought forward for presentation at the meetings of this Department, that no opportunity was afforded the Deputation of accomplishing the wishes of the Council; and the occasion was, therefore, unfortunately lost of enlisting the sympathy and obtaining the cooperation of this extensive and influential Association in the promotion and investigation of Epidemiological Science.

I have, in conclusion, only to express a hope that, as we have hitherto, notwithstanding the untoward events of war and increased taxation as its consequence, steadily progressed in our work, we may still go on and prosper; and by a supply of good papers and the judicious discussion of them, as well as by the labours of our Committees, keep up our claims, on the score of our usefulness, to the support, intellectual and pecuniary, of the profession and of the public.

